# A Library of *Plasmodium vivax* Recombinant Merozoite Proteins Reveals New Vaccine Candidates and Protein-Protein Interactions

**DOI:** 10.1371/journal.pntd.0004264

**Published:** 2015-12-23

**Authors:** Jessica B. Hostetler, Sumana Sharma, S. Josefin Bartholdson, Gavin J. Wright, Rick M. Fairhurst, Julian C. Rayner

**Affiliations:** 1 Laboratory of Malaria and Vector Research, National Institute of Allergy and Infectious Diseases, National Institutes of Health, Bethesda, Maryland, United States of America; 2 Malaria Programme, Wellcome Trust Sanger Institute, Wellcome Genome Campus, Hinxton, Cambridge, United Kingdom; 3 Cell Surface Signalling Laboratory, Wellcome Trust Sanger Institute, Wellcome Genome Campus, Hinxton, Cambridge, United Kingdom; Walter and Eliza Hall Institute, AUSTRALIA

## Abstract

**Background:**

A vaccine targeting *Plasmodium vivax* will be an essential component of any comprehensive malaria elimination program, but major gaps in our understanding of *P*. *vivax* biology, including the protein-protein interactions that mediate merozoite invasion of reticulocytes, hinder the search for candidate antigens. Only one ligand-receptor interaction has been identified, that between *P*. *vivax* Duffy Binding Protein (PvDBP) and the erythrocyte Duffy Antigen Receptor for Chemokines (DARC), and strain-specific immune responses to PvDBP make it a complex vaccine target. To broaden the repertoire of potential *P*. *vivax* merozoite-stage vaccine targets, we exploited a recent breakthrough in expressing full-length ectodomains of *Plasmodium* proteins in a functionally-active form in mammalian cells and initiated a large-scale study of *P*. *vivax* merozoite proteins that are potentially involved in reticulocyte binding and invasion.

**Methodology/Principal Findings:**

We selected 39 *P*. *vivax* proteins that are predicted to localize to the merozoite surface or invasive secretory organelles, some of which show homology to *P*. *falciparum* vaccine candidates. Of these, we were able to express 37 full-length protein ectodomains in a mammalian expression system, which has been previously used to express *P*. *falciparum* invasion ligands such as PfRH5. To establish whether the expressed proteins were correctly folded, we assessed whether they were recognized by antibodies from Cambodian patients with acute vivax malaria. IgG from these samples showed at least a two-fold change in reactivity over naïve controls in 27 of 34 antigens tested, and the majority showed heat-labile IgG immunoreactivity, suggesting the presence of conformation-sensitive epitopes and native tertiary protein structures. Using a method specifically designed to detect low-affinity, extracellular protein-protein interactions, we confirmed a predicted interaction between *P*. *vivax* 6-cysteine proteins P12 and P41, further suggesting that the proteins are natively folded and functional. This screen also identified two novel protein-protein interactions, between P12 and PVX_110945, and between MSP3.10 and MSP7.1, the latter of which was confirmed by surface plasmon resonance.

**Conclusions/Significance:**

We produced a new library of recombinant full-length *P*. *vivax* ectodomains, established that the majority of them contain tertiary structure, and used them to identify predicted and novel protein-protein interactions. As well as identifying new interactions for further biological studies, this library will be useful in identifying *P*. *vivax* proteins with vaccine potential, and studying *P*. *vivax* malaria pathogenesis and immunity.

**Trial Registration:**

ClinicalTrials.gov NCT00663546

## Author Summary

*Plasmodium vivax* causes malaria in millions of people each year, primarily in Southeast Asia and Central and South America. *P*. *vivax* has a dormant liver stage, which can lead to disease recurrence in infected individuals even in the absence of mosquito transmission. The development of vaccines that target blood-stage *P*. *vivax* parasites is therefore likely to be an essential component of any worldwide effort to eradicate malaria. Studying *P*. *vivax* is very difficult as this parasite grows poorly in the laboratory and invades only small numbers of young red blood cells in patients. Due to these and other challenges, only a handful of *P*. *vivax* proteins have been tested as potential vaccines. To generate more vaccine candidates, we expressed the entire ectodomains of 37 proteins that are predicted to be involved in *P*. *vivax* invasion of red blood cells. Antibodies from Cambodian patients with *P*. *vivax* malaria recognized heat-sensitive epitopes in the majority of these proteins, suggesting that they are natively folded. We also used the proteins to screen for both predicted and novel protein-protein interactions, confirming that the proteins are functional and further supporting their potential as vaccine candidates. As a new community resource, this *P*. *vivax* recombinant protein library will facilitate future studies of *P*. *vivax* pathogenesis and immunity, and greatly expands the list of candidate vaccine antigens.

## Introduction

*Plasmodium vivax* causes an estimated 70–391 million cases of malaria each year [1,2], but is relatively neglected in research programs compared to the more deadly *P*. *falciparum*. While *P*. *vivax* malaria is often considered benign, this description is challenged by a growing body of evidence that the morbidity of vivax malaria significantly impacts societies and economies worldwide, and that the severity and mortality of vivax malaria are either historically unrecognized, increasing in incidence, or both [3,4]. There is currently no effective vaccine to prevent *P*. *vivax* infection, reduce the prevalence of vivax malaria, or ameliorate the severity of this disease. Given the existence of the dormant hypnozoite stage of *P*. *vivax* in the liver, which can give rise to malaria relapses in the absence of transmission, a vaccine targeting the illness-causing stages of *P*. *vivax* in the blood will be essential to worldwide malaria eradication efforts.

Major gaps in our knowledge of *P*. *vivax* biology, including critical events involved in invading and developing within red blood cells (RBCs), hinder the search for a blood-stage vaccine. In particular, our current understanding of the molecular mechanisms by which *P*. *vivax* merozoites invade reticulocytes, a process that is essential for parasite survival and therefore a potential vaccine target, is extremely limited. Only a single ligand-receptor interaction, that between *P*. *vivax* Duffy Binding Protein (PvDBP) and erythrocyte Duffy Antigen Receptor for Chemokines (DARC), has been identified to date, whereas multiple *P*. *falciparum* ligand-receptor interactions are known [5]. Likewise, natural human immune responses to *P*. *vivax* during and after infection have been the subject of only limited study. Identifying naturally-acquired, clinically-protective immune responses is an invaluable step in selecting vaccine candidates for further development. While a few small-scale studies have produced full-length *P*. *vivax* recombinant proteins, even fewer have investigated whether immune IgG from *P*. *vivax*-exposed individuals recognize these proteins [6–21]. The only two large-scale immunoreactivity screens did not exclusively use full-length proteins, and may therefore have missed critical epitopes [22,23]. A review of immunoepidemiological studies of *P*. *vivax* malaria [24] found that only three antigens [MSP1, MSP3.10 (MSP3α), and MSP9] were consistently associated with protection, underscoring the need for a much broader set of antigens for study.

Due in part to our lack of knowledge about the molecular mechanisms of merozoite invasion and antimalarial immunity, relatively few *P*. *vivax* antigens have been investigated as vaccine candidates [25–33]. PvDBP, which plays a central role in *P*. *vivax* invasion, has been the target of extensive study and is a promising candidate. The DARC-binding PvDBP region II (DBPII) domain is highly polymorphic and under immune pressure [34–37], and elicits strain-specific antibody responses [21]. While this diversity makes the development of a strain-transcending vaccine a complex task, strategies to overcome this challenge are being actively pursued, most notably through the use of a synthetic DBPII-based vaccine candidate, termed DEKnull, which lacks an immunodominant variant epitope [38,39]. Numerous recent studies have observed low levels of *P*. *vivax* infection in Duffy-negative individuals in Africa and South America [40–47], although this does not necessarily mean that PvDBP plays no role in invasion. In fact, initial sequencing data of circulating *P*. *vivax* strains in Madagascar, where Duffy-negative infectivity is most common, revealed a highly prevalent PvDBP duplication [48]. However, while PvDBP remains a high-priority target, these complicating factors and previous experience with monovalent *P*. *falciparum* blood-stage vaccines, which thus far have not provided protection, suggest that a multivalent vaccine that generates cumulative immune responses should be explored. For such a multi-target *P*. *vivax* vaccine to be generated, a comprehensive and systematic approach is needed to better understand *P*. *vivax* invasion and identify additional vaccine candidates.

To address this need, we generated a library of full-length *P*. *vivax* ectodomains from merozoite proteins that are potentially involved in recognizing and invading reticulocytes, and explored their immunoreactivity and function. We screened the library against pooled plasma from Cambodian patients with acute vivax malaria, and used a systematic protein interaction screening assay to identify interactions between proteins within the library, which confirmed one predicted protein-protein interaction and identified two new interactions. Using this library, we aim to significantly enhance our understanding of *P*. *vivax* malaria pathogenesis and immunity, expand the list of blood-stage vaccine candidates, and provide a useful resource for the *P*. *vivax* research community. Our library of expression plasmids is now freely available through the non-profit plasmid repository, Addgene (www.addgene.org).

## Methods

### Ethics statement

Adults or the parents of children provided written informed consent under a protocol approved by the National Ethics Committee for Health Research in Cambodia and the NIAID Institutional Review Board in the United States (ClinicalTrials.gov Identifier, NCT00663546).

### Collection of *P*. *vivax*-exposed plasma

In Pursat Province, Western Cambodia, we obtained 17 plasma samples from 14 patients experiencing their third, fourth, or fifth episode of acute vivax malaria within 2 years of their initial episode on our clinical protocol (S1 Table). Control sera from five malaria-naïve individuals were obtained from Interstate Blood Bank (Memphis, TN, USA). Equal volumes of individual plasma or serum samples were pooled for use in ELISA.

### Selection and expression of *P*. *vivax* merozoite proteins

Expression plasmids corresponding to the entire ectodomains of secreted and membrane-embedded *P*. *vivax* merozoite proteins were designed and constructed essentially as previously described [49,50]. Briefly, the entire ectodomain protein sequences were identified by removing the endogenous signal peptide, transmembrane domain, and glycosylphosphatidylinositol (GPI) anchor sequences (if present), and the corresponding nucleic acid sequences were codon-optimized for expression in human cells. N-linked glycosylation sequons were mutated from NXS/T to NXA (where X is any amino acid except proline) to prevent glycosylation when proteins were expressed in human cells. The final constructs were chemically synthesized and sub-cloned into a derivative of the pTT3 expression vector [51], which contains an N-terminal signal peptide, a C-terminal rat Cd4 domain 3 and 4 (Cd4d3+d4) tag, and biotinylatable peptide using flanking NotI and AscI restriction sites (Geneart AG, Germany). All GPI anchors were predicted by Carlton *et al*. using both GPI-HMM and manual inspection of *P*. *vivax* orthologs of *P*. *falciparum* GPI-anchored proteins (Supplementary Information in [52]), and several of these were additionally supported by localization to the merozoite surface [53–59]. Predicted GPI anchor sequences were independently checked for a C-terminal hydrophobic region using TMHMM [60] and GPI prediction software (http://mendel.imp.ac.at/gpi/cgi-bin/gpi_pred.cgi) [61]. In the AVEXIS screens described below, these biotinylated proteins are referred to as “baits.” Beta-lactamase-tagged, “prey”-expressing plasmids were produced by subcloning each insert into a plasmid containing a pentamerization domain conjugated to the beta-lactamase tag as described [49,50]. All biotinylatable bait expression plasmids are available from the non-profit plasmid repository, Addgene (www.addgene.org).

*P*. *vivax* biotinylated bait and beta-lactamase-tagged prey recombinant proteins were expressed in HEK293E cells as described [49,50]. Briefly, HEK293E cells were maintained with shaking in Freestyle 293 Expression Medium (Invitrogen, USA) supplemented with G418 (50 mg/l), pen/strep (10000 units/l), and heat-inactivated fetal bovine serum (1%) at 37°C in a 5% CO_2_ atmosphere. Bait proteins were enzymatically biotinylated during synthesis using media supplemented with D-biotin (100 μM) and were co-transfected with a biotin ligase expression plasmid expressing a secreted BirA protein, along with the bait construct in a 1:10 ratio. Bait and prey plasmids were transiently transfected [49,51], and after 3–6 days, cultures were centrifuged (4000 rpm for 15 min) and supernatants passed through a 0.2-μm filter. Bait proteins were dialyzed in HBS to remove excess D-biotin. Cultures were stored in 10 mM sodium azide at 4°C until use.

### Enzyme-linked immunosorbent assay (ELISA)

Biotinylated bait proteins were immobilized on streptavidin-coated plates (Thermo Scientific Nunc, Denmark), and the dilution required to saturate all biotin binding sites was determined by ELISA as previously described [62]. Briefly, proteins were serially diluted in HBS/1% BSA for 1 h, plates were washed 3 times in HBS/0.1% Tween, incubated with mouse anti-rat Cd4 antibody (OX68, 1:1000) in HBS/1% BSA for 1 h, washed 3 times in HBS/0.1% Tween, incubated with goat anti-mouse alkaline phosphatase antibody (1:5000, Sigma, USA) in HBS/1% BSA for 1 h, washed 3 times in HBS/0.1% Tween, and washed once in HBS. Proteins were then incubated with phosphate substrate (1 mg/ml, Sigma) in either diethanolamine buffer (10% diethanolamine, 0.5 mM magnesium chloride, pH 9.2) or coating buffer (0.015 M sodium carbonate, 0.035 M sodium bicarbonate, pH 9.6) and detected by measuring absorbance at 405 nm. Each protein was concentrated or diluted using Vivaspin 30-kDa spin columns (Sartorius, Germany) or HBS/1% BSA, respectively. As an approximate guide, the biotin binding sites are saturated at a biotinylated protein concentration of 0.3–0.5 μg/ml [62,63]. Based on this, a categorization of approximate protein expression levels could be made between “high” (likely > 5 μg/ml) for those proteins that could be diluted more than 1:10 and still saturate biotin binding sites, “medium” (approximately 0.5–5 μg/ml) for those proteins using 1:1 through 1:10 dilutions, and “low” (likely < 0.5 μg/ml) for those proteins that showed some signal, but failed to saturate the biotin binding sites over a range of dilutions prior to concentrating. These categories should be treated only as a guide for future studies since, due to expected experimental variation in transient transfection efficiency, we observed significant batch-to-batch variation in expression.

We performed ELISAs to assess the seroreactivity and conformation of the *P*. *vivax* recombinant proteins. *P*. *vivax* biotinylated proteins were denatured at 80°C for 10 min or left untreated, and then immobilized on streptavidin-coated plates as above. Proteins were incubated in a single assay in triplicate wells with pooled plasma (1:600 or 1:1000) from 14 vivax malaria patients from Cambodia or pooled serum from five malaria-naïve individuals from the United States in HBS/1% BSA. After 2 h of incubation, wells were washed 3 times with HBS/0.1% Tween, incubated with alkaline phosphatase-conjugated goat anti-human IgG (1:1000, KPL Inc., USA) in HBS/1% BSA for 2 h, washed 3 times with HBS/0.1% Tween, and washed once with HBS. Seroreactivity was detected using phosphate substrate (1 mg/ml, Sigma) in coating buffer (0.015 M sodium carbonate, 0.035 M sodium bicarbonate, pH 9.6) and measuring absorbance at 405 nm at various times up to 30 min.

### Western blotting

*P*. *vivax* biotinylated bait proteins were reduced with NuPAGE Sample Reducing Agent (Invitrogen), heated at 70°C for 10 min, fractionated by SDS-PAGE, and transferred to nitrocellulose membranes. After blocking in HBS/0.1% Tween/2% BSA at 4°C overnight, membranes were incubated with streptavidin-horseradish peroxidase (1:2000, Cell Signaling Technology, USA) in HBS/0.1% Tween/2% BSA at room temperature for 1–2 h, developed using the chemiluminescence substrate Amersham ECL Prime (GE Healthcare, USA), and exposed to X-ray film.

### Avidity-based extracellular interaction screen (AVEXIS)

*P*. *vivax* biotinylated bait and beta-lactamase-tagged prey proteins were screened using AVEXIS [49,50]. Prey proteins were first normalized using the beta-lactamase tag activity as a proxy of protein concentration by the rate of nitrocefin hydrolysis, essentially as described [62] and were concentrated or diluted as needed using Vivaspin 20- or 30-kDa spin columns (Sartorius, Germany) or HBS/1% BSA [62]. Biotin binding sites on streptavidin-coated plates (Thermo Scientific Nunc) were saturated with biotinylated bait proteins for 1 h. Plates were then washed 3 times in HBS/0.1% Tween, probed with beta-lactamase-tagged prey proteins for 1 h, washed twice with HBS/0.1% Tween, washed once with HBS, and incubated with nitrocefin (60 μl at 125 μg/ml; Calbiochem, USA). Positive interactions were indicated by nitrocefin hydrolysis, which was detected by measuring absorbance at 485 nm at 90 min.

### Surface plasmon resonance (SPR)

The entire ectodomains of P12, P41, and MSP7.1 were sub-cloned into a modified plasmid containing a 6-His tag [49], expressed as above, and purified by immobilized metal ion affinity chromatography using HisTrap HP columns on an AKTA Xpress (GE Healthcare) following the manufacturer’s instructions. Purified proteins were subjected to size-exclusion chromatography (SEC) by filtering through either a Superdex 200 Increase 10/300 GL column or Superdex 200 Tricorn 10/600 GL column in HBS-EP (HBS, 3 mM EDTA, 0.005% v/v surfactant P20) using an AKTA Xpress (GE Healthcare) immediately before use, as described [64]. Extinction coefficients were calculated using the protein sequence and the ProtParam tool (http://www.expasy.org/tools/protparam.html). SPR was performed using a Biacore T100 instrument (GE Healthcare) at 37°C in HBS-EP running buffer. Biotinylated P12, P41, MSP3.10, and PVX_110945 proteins (expressed as above) were immobilized to the sensor chip using the Biotin CAPture Kit (GE Healthcare) in approximately molar equivalents with a negative control reference biotinylated rat Cd4d3+4 (tag alone). Increasing concentrations of each soluble purified analyte was injected at low (20 μl/min) flow rates for equilibrium experiments. The chip was regenerated between each injection cycle. Biacore T100 evaluation software version 2.0.3 (GE Healthcare) was used to analyze the reference-subtracted sensorgrams. Binding was investigated by plotting the maximum binding response for a range of concentrations (R_eq_) prior to washing, and plotted as a function of analyte concentration (C) and fitted using the equation *R*_*eq*_ = *CR*_*max*_/(*C*+*K*_*D*_), where *R*_*max*_ is the maximum binding response and *K*_*D*_ is the equilibrium dissociation constant.

## Results

### Multiple diverse *P*. *vivax* merozoite proteins are expressed in HEK293E cells

Potential *P*. *vivax* invasion-blocking vaccine candidates were selected using existing microarray data [65,66] and homology comparisons between *P*. *vivax* and *P*. *falciparum* protein sequences [67,68]. A library of 39 *P*. *vivax* merozoite proteins that are predicted to localize to the merozoite surface, micronemes, or rhoptries, or whose transcripts are most abundant during schizogony, or both, were identified and constructs for their expression in HEK293E cells designed and synthesized (see Methods). These 39 proteins were subdivided into four groups (Table 1).

**Table 1:** *P*. *vivax* recombinant merozoite proteins.

Group	Accession number	Common name	Product	Length (aa)	Expected size (kDa)	Region expressed	*P*. *falciparum* 3D7 homologs or ortholog group (OG)	Subcellular location	Expression level
**Merozoite surface proteins (MSPs)** ^1^	PVX_099980	MSP1	merozoite surface protein 1	1702	215	E20-P1721	PF3D7_0930300	Merozoite surface (GPI-anchor) [53,54]	low
	PVX_097670	MSP3.1, MSP3A^2^, MSP3γ	merozoite surface protein 3	825	114	N21-K845	None^7^		high
	PVX_097680	MSP3.3, MSP3C^2^, MSP3β	merozoite surface protein 3	997	133	D20-K1016	None^7^	Merozoite surface (no anchor) [69]	high
	PVX_097685	MSP3.4, MSP3D1	merozoite surface protein 3	1091	140	N21-M1111	OG5_126854		high
	PVX_097720	MSP3.10, MSP3H^2^, MSP3α	merozoite surface protein 3	829	113	E24-W852	None^7^	Merozoite surface (no anchor) [69]	med
	PVX_003775	MSP4^2^	merozoite surface protein 4, putative	202	45	A26-S227	PF3D7_0207000	Merozoite surface (GPI-anchor) [59]	med
	PVX_003770	MSP5^2^	merozoite surface protein 5	346	62	R22-S367	PF3D7_0206900>	Microneme and or/Apical (GPI-anchor) [59]	high
	PVX_082700>	MSP7.1^3^	merozoite surface protein 7 (MSP7)	399	70	E22-Y420	None		low
	PVX_082675	MSP7.6^3^	merozoite surface protein 7 (MSP7)	428	72	A26-N453	PF3D7_1334300		med
	PVX_082655	MSP7.9^3^	merozoite surface protein 7 (MSP7), putative	364	65	E24-V387	OG5_151950	Merozoite surface (no anchor)^8^ [70,71]	low
	PVX_114145	MSP10^2^	merozoite surface protein 10, putative	435	72	A23-S457	PF3D7_0620400	Merozoite surface (GPI-anchor) [55,56,58]	high
**6-cysteine proteins**	PVX_113775>	P12	6-cysteine protein	316	61	F24-A339	PF3D7_0612700	Merozoite surface (GPI-anchor) [56], Rhoptry [57]	high
	PVX_113780	P12p^2^	6-cysteine protein	395	68	V24-P418	PF3D7_0612800		med
	PVX_097960	P38^2^	6-cysteine protein	306	60	K29-G334	PF3D7_0508000	Merozoite surface and/or Apical (GPI-anchor)^8^ [72]	low
	PVX_000995	P41	6-cysteine protein	363	67	E22-E384	PF3D7_0404900	Merozoite surface (no anchor) [73]	med
	PVX_115165	P92	6-cysteine protein	833	118	D23-H855	PF3D7_1364100	Merozoite surface (GPI-anchor)^8^ [72]	low
**Merozoite proteins not in other families**	PVX_001725	RON12	rhoptry neck protein 12, putative	274	54	L25-S298	PF3D7_1017100	Rhoptry^8^ [74]	med
	PVX_088910	GAMA	GPI-anchored micronemal antigen, putative	729	103	L21-S749	PF3D7_0828800	Merozoite surface (GPI-Anchor)^8^ [75]	high
	PVX_090075	Pv34^2^	apical merozoite protein^2^	323	61	N25-S347	PF3D7_0419700	Rhoptry (GPI-anchor)^8^ [76]	high
	PVX_090210	ARP	asparagine-rich protein	265	54	K21-P285	PF3D7_0423400	Rhoptry^8^ [77]	high
	PVX_090240	CyRPA	cysteine-rich protective antigen, putative	344	65	T23-D366	PF3D7_0423800	Apical^8^ [78]	med
	PVX_095055	RIPR	Rh5 interacting protein, putative	1054	144	N22-A1075	PF3D7_0323400>	Microneme and merozoite surface^8^ [79]	very low
	PVX_098712	RhopH3	high molecular weight rhoptry protein 3, putative	866	125	R25-T890	PF3D7_0905400	Rhoptry	low
	PVX_110810	DBP	Duffy receptor precursor	986	135	V23-T1008	OG5_148188	Microneme [59,80]	low
		DBP-RII	Duffy binding protein region II	328	64	D194-T521			low
	PVX_111290	MTRAP	merozoite TRAP-like protein, putative	292	58	K24-G315	PF3D7_1028700	Microneme^8^ [81]	high
	PVX_121885	CLAG^4^	cytoadherence linked asexual protein, CLAG, putative	1138	159	Y25-L1162	OG5_138272	Rhoptry^8^	very low
	PVX_081550		StAR-related lipid transfer protein, putative	473	80	R23-F495	PF3D7_0104200		low
	PVX_084815		conserved *Plasmodium* protein, unknown function^2^	249	53	R22-A270^6^	PF3D7_1434400	GPI-anchor [52]	very low
	PVX_084970		conserved *Plasmodium* protein, unknown function^2^	892	121	D24-S915	PF3D7_1431400	GPI-anchor [52]	low
	PVX_116775		conserved *Plasmodium* protein, unknown function^2^	314	60	E21-S334	PF3D7_1321900		low
**No known *P*. *falciparum* 3D7 homologs**	PVX_101590	RBP2-like^4^	reticulocyte-binding protein 2 (RBP2), like	619	97	K22-K640	None		med
	PVX_001015		6-cysteine protein, putative^5^	330	62	Q28-T357	None	GPI-anchor [52]	low
	PVX_110945		hypothetical protein	316	61	D28-K343	None		low
	PVX_110950		conserved *Plasmodium* protein, unknown function^2^	379	66	K24-L402	None		low
	PVX_110960		hypothetical protein	648	92	A23-K670	None		low
	PVX_110965		conserved *Plasmodium* protein, unknown function^2^	421	69	D21-A441	None		low

Proteins and their GeneDB accession numbers are classified into four groups, based on whether they are members of the MSP family, the 6-cysteine family, neither family and have a known *P*. *falciparum* homolog, or neither family and have no known *P*. *falciparum* homolog. Common names and products are listed based on existing *P*. *vivax* annotation. The expected size includes a ~25-kDa C-terminal rat Cd4d3+d4 tag. Boundaries of full-length ectodomains are delimited by the N- and C-terminal amino acid residues according to their codon positions along the *P*. *vivax* Sal1 reference protein sequence. *P*. *falciparum* accession numbers are listed when only one homolog exists and OrthoMCL clusters are listed when multiple homologs exist. Subcellular locations are based on existing *P*. *vivax* literature or predictions based on existing *P*. *falciparum* literature where noted. The presence of a glycosylphosphatidylinositol (GPI) anchor sequence is indicated [52]. Expression levels are based on an estimated 0.3–0.5 μg/ml required to saturate biotin binding sites on plates [62,63]. Levels are listed as a guide, as significant batch-to-batch variability was observed. Groups include “high” (> 5 μg/ml), “med” for medium (0.5–5 μg/ml), and “low” (< 0.5 μg/ml) expressors, as defined in Methods.

^1^MSP9 and MSP1P failed to be sub-cloned or expressed, respectively

^2^Based on genedb.org annotation

^3^Based on MSP7 family naming in [70]

^4^Based on *P*. *vivax* product description

^5^6-cysteine protein upstream of P52 and P36 in *P*. *vivax* but not *P*. *falciparum*

^6^S270 in unmodified sequence

^7^ *P*. *vivax* and *P*. *falciparum* MSP3s have the same numeric designation, but do not appear to be orthologs [82] or fall into the same OrthoMCL clusters

^8^Based on localization in *P*. *falciparum*

#### Merozoite surface proteins

Merozoite surface proteins (MSPs) are primarily exposed on the plasma membrane of the invasive merozoite, and several *P*. *falciparum* MSPs have been tested as vaccine candidates. We selected a total of 13 MSPs, eight of which have known subcellular localizations in *P*. *vivax* [53–56,58,59,69,83,84] that are similar to those of their *P*. *falciparum* homologs—except MSP5, which has a microneme and/or apical distribution in *P*. *vivax* but a merozoite surface distribution in *P*. *falciparum* [59]. GPI anchor sequences were predicted for MSP5 [52] and both predicted and supported by merozoite surface localization experiments in *P*. *vivax* for MSP1, MSP4, and MSP10 [52–56,58,59]. GPI anchors were experimentally confirmed in *P*. *falciparum* homologs for MSP1, MSP4, and MSP5 [72]. *P*. *vivax* has 12 members of the MSP3 family, which are not clear homologues of the *P*. *falciparum* MSP3 family but may serve similar functions [82]; four representative MSP3s were selected for expression in this library. Similarly three MSP7s were selected to represent the 11 MSP7 family members, which Kadekoppala and Holder [70] have named sequentially in the order they occur on chromosome 12, beginning with PVX_082700 as MSP7.1 and ending with PVX_082650 as MSP7.11. Despite repeated attempts, we were unable to express the MSP1 paralog, MSP1P, or to sub-clone MSP9 into the expression vector.

#### 6-cysteine proteins

6-cysteine proteins are defined by a characteristic arrangement of cysteine residues and are expressed at multiple stages of the *P*. *falciparum* life cycle; several members are considered stage-specific *P*. *falciparum* vaccine candidates. We selected five of 13 *P*. *vivax* 6-cysteine proteins, four of which are most homologous to the *P*. *falciparum* proteins known to be involved in blood-stage parasite development [72]. Transcriptional data suggest that P12p may also be involved in the blood-stage development of *P*. *berghei* parasites [85]. GPI anchors were predicted for three proteins (P12, P38, and P92) [52], all of which were experimentally confirmed in *P*. *falciparum* orthologs [72]. The predicted GPI anchor in P12 is supported by a merozoite surface and/or rhoptry localization [56,57].

#### Merozoite proteins not in other families

Twenty other proteins, which do not belong to the aforementioned *P*. *vivax* MSP or 6-cysteine protein families, were selected because they are known or suspected to localize to the merozoite surface, micronemes, or rhoptries. Localization to these regions is predicted based on *P*. *falciparum* homologs [74–79,81] and several have predicted GPI anchors (GAMA, Pv34, PVX_084815, PVX_084970, and PVX_001015) [52]. These 20 proteins fall into two groups, with 14 (including PvDBP) having a *P*. *falciparum* homolog or ortholog, and six having neither. The latter six proteins include a reticulocyte binding protein (RBP2-like, PVX_101590) that is much smaller than other RBPs, which are typically > 250 kDa and therefore unlikely to express well in the HEK293E cell system; a hypothetical 6-cysteine protein (PVX_001015) that is located upstream from the 6-cysteine proteins P52 and P36 at a microsyntenic breakpoint on *P*. *vivax* chromosome 3, and has a predicted GPI-anchor [52]; and four hypothetical proteins [86] that are syntenic to an MSP-encoding region in *P*. *falciparum* but have no *P*. *falciparum* orthologs. Little is known about the hypothetical proteins in our library, except that their transcripts are most abundant in early-to-late schizonts [66] and one of them (PVX_110960) contains a merozoite SPAM domain (Pfam accession number, PF07133).

**Fig 1:**
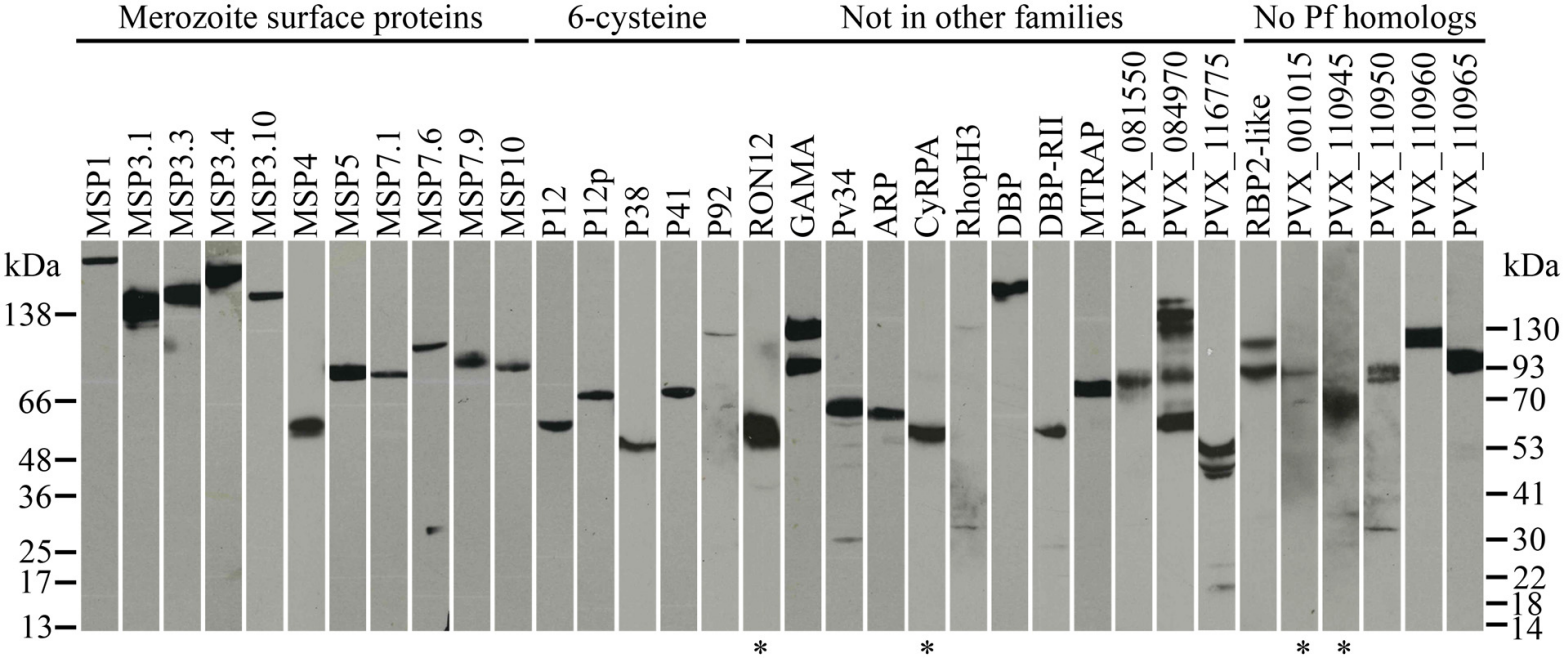
Western blot analysis confirms expression of 34/37 *P*. *vivax* recombinant proteins. Biotinylated proteins were resolved by SDS-PAGE under reducing conditions, blotted, and probed using streptavidin-HRP. All proteins contain a ~25-kDa rat Cd4d3+4 tag. (*) indicates proteins that were run with the right ladder; all others were run with the left or both ladders.

The expression of 95% (37/39) of the biotinylated full-length protein ectodomains was confirmed by an ELISA that detected the rat Cd4d3+d4 tag; 34 of these proteins were also detected by western blot analysis (Fig 1). The sizes of expressed proteins ranged from 45 to 215 kDa (which includes the ~25-kDa rat Cd4d3+d4 tag), and generally conform to predicted sizes (Table 1). Several proteins, such as GAMA and RBP2-like, showed multiple bands suggesting they were proteolytically processed, as was seen for some recombinant proteins in an analogous *P*. *falciparum* library [50]. Protein expression varied widely, with 44% (17/39) of the *P*. *vivax* bait proteins expressing well at over 0.5 μg/ml (see “high” and “medium” in Methods). An additional 51% (20/39) of proteins had low expression (< 0.5 μg/ml), but most could be effectively concentrated and used in downstream screens. Three proteins, CLAG, RIPR, and PVX_084815, showed very low expression and were detected by ELISA but not western blot.

### *P*. *vivax* recombinant proteins are immunoreactive and contain conformational epitopes

**Fig 2:**
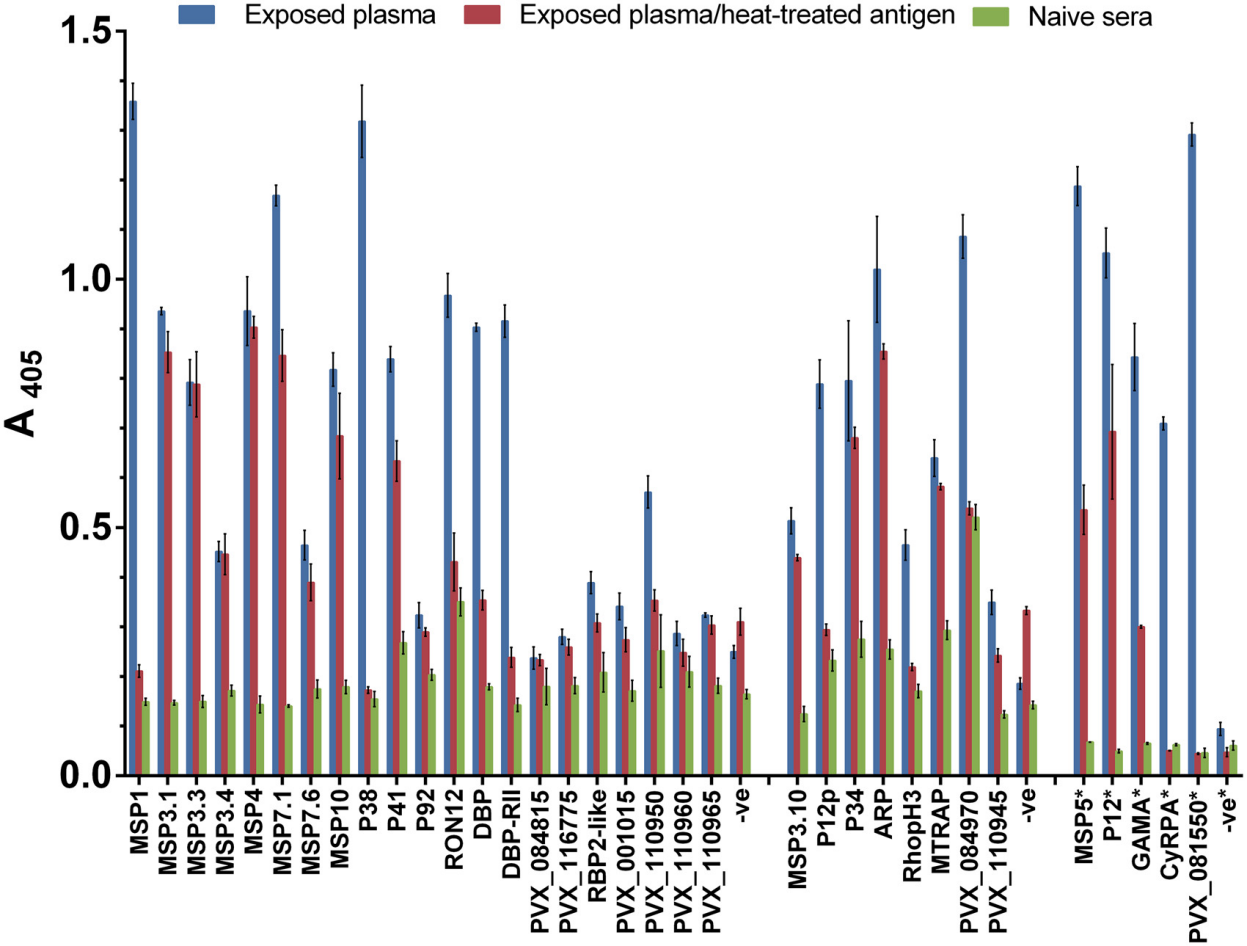
Multiple *P*. *vivax* recombinant proteins are immunoreactive and contain conformational epitopes. The immunoreactivity of 34 biotinylated *P*. *vivax* recombinant proteins was assessed using diluted (1:600) plasma pools from 14 Cambodian vivax malaria patients (blue bars) and five American malaria-naïve individuals (green bars). The immunoreactivity of heat-treated proteins was assessed in parallel using the Cambodian plasma pool (red bars); reduced responses indicate the presence of heat-labile conformational epitopes. The immunoreactivity of highly reactive proteins (*) was assessed using more-diluted (1:1000) plasma pools. Absorbance (A) at 405 nm was measured at various times, but only the mean value nearest to 1.0 for each antigen is shown. Negative control (–ve) was rat Cd4d3+d4 tag. Bar charts show mean ± SD; n = 3.

To test whether the biotinylated *P*. *vivax* recombinant protein ectodomains were immunoreactive, and to establish whether they contained conformational epitopes, we screened them by ELISA against diluted (1:600) pooled plasma from 14 patients with acute vivax malaria in Cambodia and pooled control sera from five malaria-naïve individuals in the United States (Fig 2). Exposed IgG reacted more strongly than naïve IgG to all *P*. *vivax* proteins, and reacted only weakly to the rat Cd4d3+4 tag present in all expressed proteins. Five proteins (MSP5, P12, GAMA, CyRPA, and PVX_081550) were particularly reactive; thus, ELISAs for these proteins were repeated using more-diluted (1:1000) plasma pools (Fig 2). Of the 34 proteins tested, 27 showed at least a two-fold change in seroreactivity between the naïve IgG versus the exposed IgG for each protein, with seven proteins (P92, PVX_084815, PVX_116775, RBP2-like, PVX_001015, PVX_110960, and PVX_110965) showing a lesser change. The majority of proteins showed at least a three-fold change (MSP1, MSP3.1, MSP3.3, MSP3.10, MSP4, MSP5, MSP7.1, MSP10, P12, P12p, P38, P41, GAMA, ARP, CyRPA, DBP, DBP-RII, and PVX_081550).

To test whether IgG responses were directed at conformational epitopes, we heat treated all 34 proteins and screened them for seroreactivity in parallel with untreated proteins. Of the 34 proteins tested, 18 (MSP1, MSP7.1, P38, P41, RON12, DBP, DBP-RII, RBP2-like, PVX_110950, P12p, RhopH3, PVX_084970, PVX_110945, MSP5, P12, GAMA, CyRPA, and PVX_081550) showed at least a 20% decrease in seroreactivity when heat treated (Fig 2), indicating they contained conformation-sensitive epitopes, and suggesting that the recombinant proteins were properly folded. Twelve of these proteins (MSP1, P38, RON12, DBP, DBP-RII, P12p, RhopH3, PVX_084970, MSP5, GAMA, CyRPA, and PVX_081550) showed at least a 50% reduction in seroreactivity when heat treated (Fig 2). Naïve IgG showed appreciable reactivity to PVX_084970; screening this protein against individual plasma samples may resolve whether it non-specifically reacts to IgG from all or only some of the five serum donors.

### AVEXIS and SPR detect known and novel interactions between *P*. *vivax* recombinant proteins

**Fig 3:**
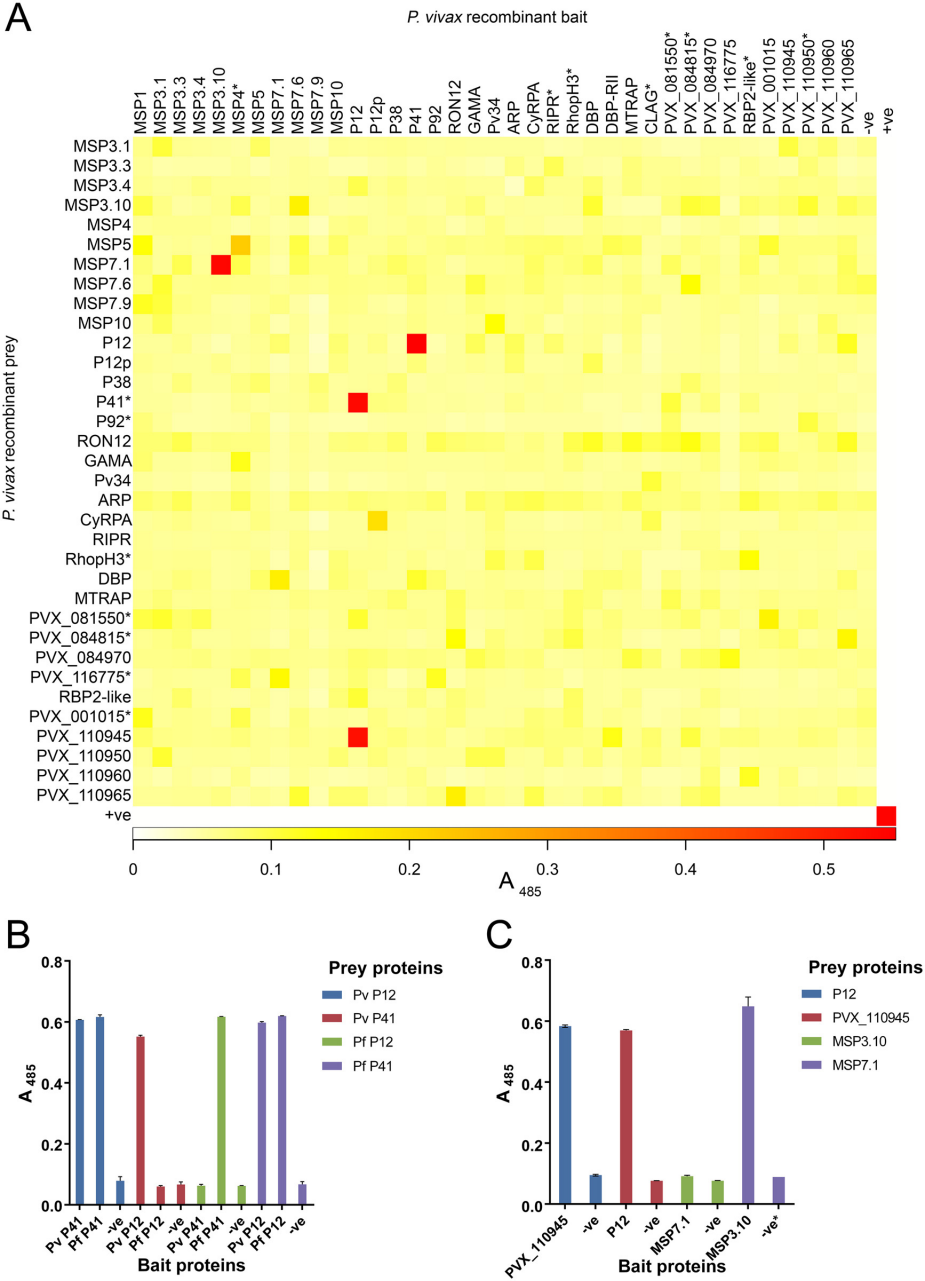
AVEXIS reveals novel interactions involving *P*. *vivax* recombinant proteins. (A) Heat map of the initial *P*. *vivax* intra-library AVEXIS, with the intensity of absorbance (A) values at 485 nm and positive putative interactions in red: P12-P41 (bait-prey and prey-bait orientations), P12-PVX_110945 (bait-prey orientation), and MSP3.10-MSP7.1 (bait-prey orientation). (*) indicates baits with low protein levels (< 0.5 μg/ml after concentrating) and preys with activity below the threshold required by the assay. Positive control (+ve) is the *P*. *falciparum* P12-P41 interaction. Negative controls (–ve) are rat Cd4d3+d4 tag in (A-C). (B) P12-P41 interaction within and between *P*. *vivax* (Pv) and *P*. *falciparum* (Pf) proteins by AVEXIS. An interaction between Pv P12-Pv P41 and Pv P12-Pf P41 in both bait and prey orientations. A, absorbance in (B-C). Bar chart shows mean with range; n = 2 in (B-C). (*) indicates n = 1. (C) Replicated *P*. *vivax* intra-library AVEXIS using re-synthesized PVX_110945 and MSP7.1 bait proteins, confirming the P12-PVX_110945 interactions in both orientations, and the MSP3.10-MSP7.1 interaction in only the bait-prey orientation.

Reflecting the general lack of knowledge about *P*. *vivax* biology, most of the recombinant proteins in our library have no known function, making it impossible to establish whether they recapitulate the function of native proteins. In some cases, however, protein-protein interactions between members of the library are either known or predicted based on homology to *P*. *falciparum*. We therefore performed a protein-protein interaction screen using the AVEXIS technology that was previously used to identify novel ligand-receptor interactions in *P*. *falciparum* [87,88]. AVEXIS consists of testing for interactions between “bait” proteins, which are biotinylated and captured by the streptavidin-coated wells of a 96-well plate, and “prey” proteins, which are enzymatically tagged and contain a pentamerization domain to increase interaction avidity. Of the 37 constructs that expressed successfully in the bait vector, 34 were successfully sub-cloned and expressed in the prey vector, though several had low expression or activity (indicated by asterisks in Fig 3A). AVEXIS was then performed using all 37 bait and 34 prey *P*. *vivax* proteins.

This intra-library AVEXIS (Fig 3A) identified three *P*. *vivax* protein-protein interactions in the bait-prey orientation: P12-P41, P12-PVX_110945, and MSP3.10-MSP7.1. The P12-P41 interaction was predicted, as the *P*. *falciparum* homologues of these proteins were recently shown to form a heterodimer [64]. The P12-P41 interaction was also detected in the reciprocal prey-bait orientation (Fig 3A). While interaction between *P*. *vivax* P12 and P41 is predicted based on the function of the *P*. *falciparum* homologues, this is the first time it has been confirmed, implying that the ability of these proteins to interact is essential for their functional activity and predates the evolutionary divergence of *P*. *falciparum* and *P*. *vivax*. To test whether specific amino acid components of the interaction have been conserved over the long evolutionary timeframe since the *P*. *falciparum* and *P*. *vivax* divergence, we tested the ability of *P*. *vivax* and *P*. *falciparum* P12 and P41 to interact with each other. *P*. *vivax* P12 and *P*. *falciparum* P41 interacted, suggesting that conserved amino acid contacts do exist (Fig 3B), but *P*. *falciparum* P12 and *P*. *vivax* P41 did not interact. Investigating the sequences and structures of these proteins in more detail may improve our understanding of their interactions. While the P12-P41 interaction was predicted, two novel interactions, P12-PVX_110945 and MSP3.10-MSP7.1, were also identified. Re-expression of PVX_110945 and MSP7.1 as baits instead of preys confirmed the PVX_110945-P12 interaction, but the MSP3.10-MSP7.1 interaction was not detected in the reciprocal prey-bait orientation (Fig 3C). The inability to recapitulate the MSP3.10-MSP7.1 interaction does not necessarily indicate that this interaction is biologically insignificant. Protein-protein interactions that are orientation-dependent have been reproducibly detected in other studies [49,89,90], and in this case may indicate a loss of activity when MSP3.10 is pentamerized in the prey vector.

**Fig 4:**
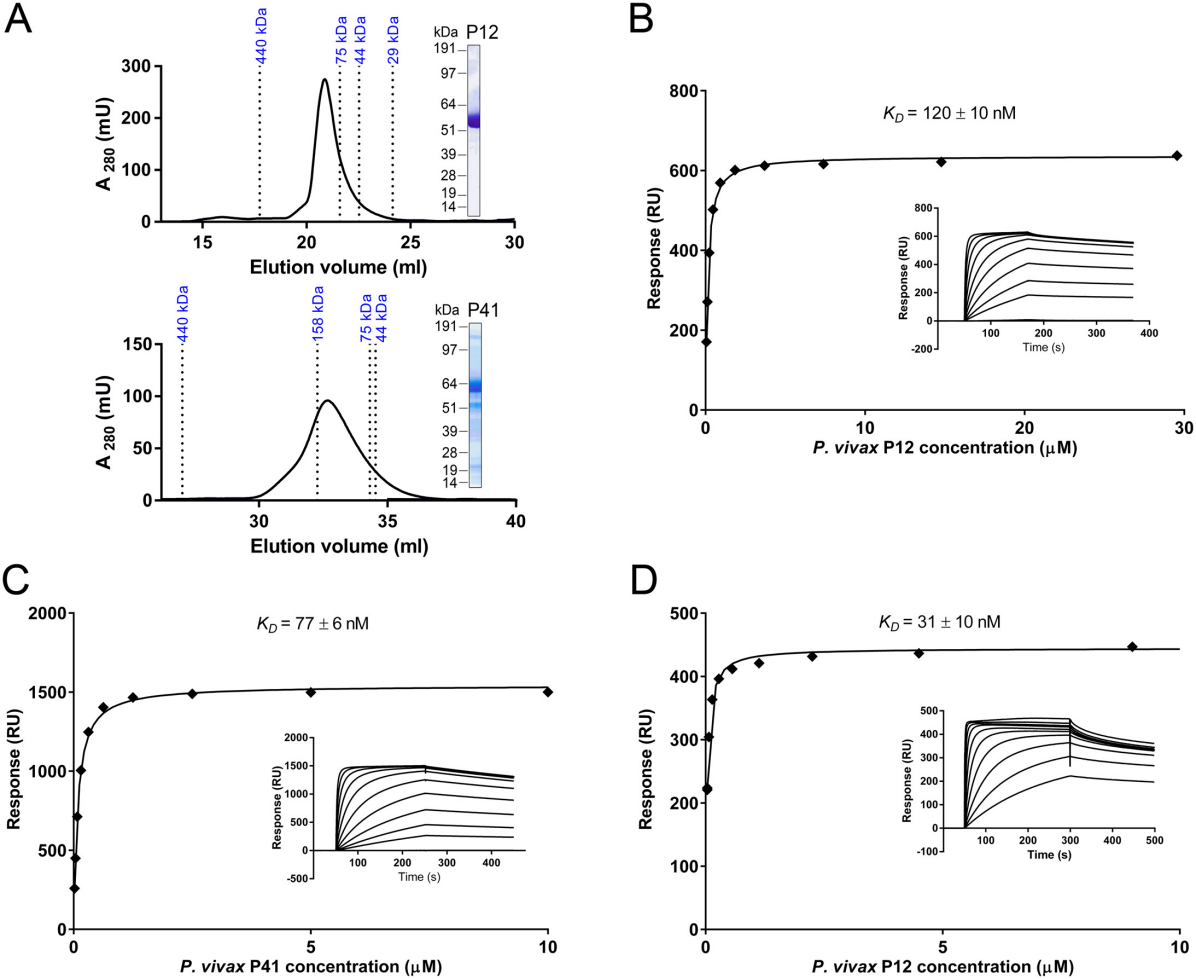
Quantification of the *P*. *vivax* P12-P41 interaction affinity by surface plasmon resonance. (A) Recombinant, his-tagged *P*. *vivax* P12 and *P*. *vivax* P41, each eluted as a monodisperse peak after SEC which resolved as a single band of the expected size by SDS-PAGE (insets). (B, C, D) Increasing concentrations of analyte protein were injected over immobilized biotinylated ligand protein. Reference-subtracted binding data were plotted as a binding curve and the equilibrium dissociation constant was calculated using *R*_eq_ = C*R*_max_/(C+*K*_D_). Experiments included analyte-ligand combinations *P*. *vivax* P12-P41 (B), *P*. *vivax* P41-P12 (C), and *P*. *vivax* P12-*P*. *falciparum* P41 (D). Lower concentrations failed to reach equilibrium, which resulted in an overestimated *K*_D_. SEC, size-exclusion chromatography.

SPR was used to validate all interactions and determine the biophysical binding parameters for the P12-P41 interaction (Fig 4). In SEC, recombinant, purified *P*. *vivax* P12 and *P*. *vivax* P41 appeared to elute at apparent molecular masses of 112 kDa and 111 kDa, which are greater than their expected sizes of 60 kDa and 66 kDa, respectively (Fig 4A). Since the apparent masses are about two times the expected sizes, this finding may indicate that these proteins form homodimers in solution, which is supported by gel data obtained under native conditions (S1 Fig). *P*. *falciparum* P12 and P41 also eluted at higher-than-expected masses of 90 kDa and 105 kDa, respectively [64]. No P12-P12 or P41-P41 self-binding was observed by SPR (S2 Fig) or AVEXIS (Fig 3A), indicating that the proteins, whether monomers or dimers, are unable to self-associate into higher-order structures. Equilibrium binding experiments between *P*. *vivax* P12-P41 showed clear evidence of saturation, thus demonstrating the interaction’s specificity (Fig 4B and 4C). However, due to limited amount of protein, several of the lower concentrations of analyte did not achieve equilibrium in both bait-prey orientations with the consequence that the calculated equilibrium dissociation constants (*K*_D_) are likely to be slightly overestimated; that is, the interaction has a higher affinity (Fig 4B and 4C). When *P*. *vivax* P12 was used as the purified his-tagged analyte and *P*. *vivax* P41 as the immobilized biotinylated ligand the *K*_D_ was 120 ± 10 nM, and when the proteins were used in the reverse orientation the *K*_D_ was 77 ± 6 nM. The *P*. *vivax* P12-P41 interaction therefore has an affinity that is at least three times higher than the *P*. *falciparum* P12-P41 interaction, with the *P*. *vivax* P12-P41 *K*_D_ < 100 nM being much lower than the *P*. *falciparum* P12-P41 *K*_D_ of 310 nM [44]. Interspecies binding detected using AVEXIS was confirmed by SPR between *P*. *vivax* P12 as the purified his-tagged analyte and *P*. *falciparum* P41 as the immobilized biotinylated ligand (Fig 4D). The *K*_D_ of 31 ± 10 nM for this interaction also suggests that it has a much higher affinity than the *P*. *falciparum* P12-P41 interaction.

**Fig 5:**
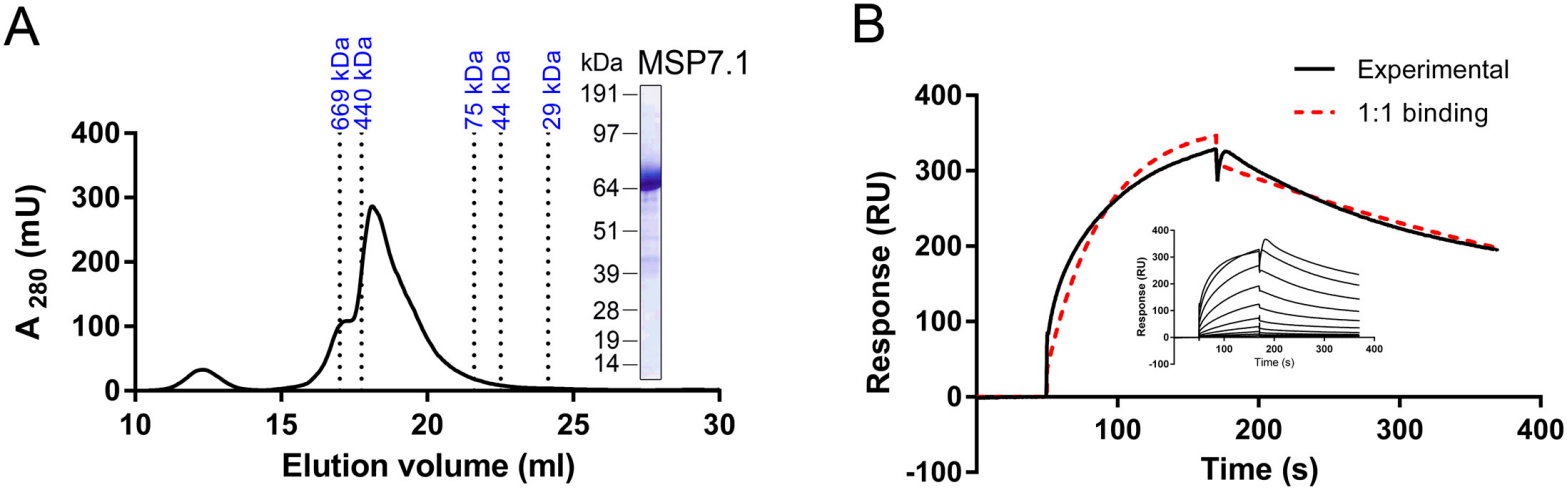
Surface plasmon resonance confirms the *P*. *vivax* MSP3.10-MSP7.1 interaction. (A) Recombinant, his-tagged *P*. *vivax* MSP7.1 eluted as a main peak with a small shoulder at higher masses than expected after SEC, likely due to oligomerization, and with a main band of the expected size by SDS-PAGE (inset). (B) Increasing concentrations of *P*. *vivax* MSP7.1 were injected over immobilized biotinylated *P*. *vivax* MSP3.10. Relatively high-affinity binding was observed, although none of the concentrations used reached equilibrium (inset). The binding did not fit a 1:1 model (red dashed line). The increase in response units at the start of the dissociation phase at the higher analyte concentrations of *P*. *vivax* MSP7.1 is likely an artefactual buffer effect. SEC, size-exclusion chromatography.

SPR was also used to study the *P*. *vivax* MSP3.10-MSP7.1 interaction (Fig 5). The recombinant, purified *P*. *vivax* MSP7.1 eluted as a main peak with evidence of higher molecular mass forms by SEC. The main peak eluted at a much larger-than-expected molecular mass of 69 kDa, suggesting oligomerization or aggregation of the protein in solution (Fig 5A), which is additionally supported by gel data obtained under native conditions (S1 Fig). Despite little sequence conservation between MSP7 family members for *P*. *vivax* and *P*. *falciparum* [70], this is also seen in *P*. *falciparum* MSP7 purifications, though with additional smaller forms present [91]. Other *Plasmodium* surface proteins, such as *P*. *falciparum* MSP2 and MSP3, are also known to form higher-order structures [92–95]. Equilibrium binding experiments using *P*. *vivax* MSP3.10 as ligand and *P*. *vivax* MSP7.1 as analyte showed a relatively high binding affinity (Fig 5B), but a *K*_D_ could not be calculated since the binding did not reach equilibrium at any of the concentrations tested. In addition, the binding did not fit a 1:1 model (Fig 5B, shown in red), most likely due to oligomerization of the purified *P*. *vivax* MSP7.1. Such complex binding behavior was also observed between P-selectin and *P*. *falciparum* MSP7, which also oligomerizes [91]. SPR was also used to explore the P12-PVX_110945 interaction identified by AVEXIS. While this confirmed an extremely weak interaction between recombinant purified *P*. *vivax* P12 as analyte and biotinylated PVX_110945 as ligand, this was not further studied (S3 Fig).

## Discussion

Biological research studies of *P*. *vivax* generally lag behind those of *P*. *falciparum*, in large part due to the lack of a robust *in vitro* culture system, which complicates investigation of *P*. *vivax* erythrocyte invasion and thus identification of *P*. *vivax* blood-stage vaccine candidates. Multiple studies have investigated single or few *P*. *vivax* merozoite proteins, but a comprehensive library of full-length ectodomains of merozoite surface, microneme, and rhoptry proteins for functional studies has not yet been assembled. To build such a library, we selected 39 proteins that are known or predicted to localize to the surface or apical organelles of merozoites based on published *P*. *vivax* and *P*. *falciparum* studies, and published microarray data showing gene upregulation during *P*. *vivax* schizogony. We were able to express 37 of these proteins as recombinant ectodomains, including several members of the MSP and 6-cysteine protein families, additional invasion-related and/or GPI-anchored proteins, as well as several proteins with no *P*. *falciparum* homologs. Our success in expressing 95% of *P*. *vivax* merozoite proteins at levels useable for biochemical studies is comparable to similar efforts at expressing *P*. *falciparum* merozoite proteins [50], and demonstrates the broad utility of the human HEK293E cell expression system in producing high-yield, high-quality proteins for *Plasmodium* research. Multiple systems have now been used to express panels of *Plasmodium* proteins [23,50,96–101], and all have their strengths and weaknesses. The wheat germ extract and *E*. *coli* in vitro protein expression systems are scalable, but lack the context of a complete eukaryotic secretory system including some post-translational modifications, though the lack of *N*-linked glycosylation is helpful in the case of *Plasmodium*. Our HEK293 system is more medium throughput, but is well suited to large proteins, may represent post-translational modifications better, and has already demonstrated its utility in immunoepidemiological and functional studies [63,64,87].

Protein expression levels were evaluated for patterns that predicted success. From previous experience using the HEK293 system, proteins larger than 250 kDa are frequently not expressed or expressed at low levels. However, size alone was not a useful predictor of expression levels in this library, as we sometimes observed high and low expression levels for large and small proteins, respectively (e.g., high expression for MSP3.4 [140 kDa], and low expression for PVX_084815 [53 kDa]). The effects of amino acid composition and predicted protein folding on expression are not yet clear, but useful predictors of expression levels may become apparent as we expand our *Plasmodium* plasmid library.

In order to maximize our chance for successful expression, we used codon-optimized, protein ectodomains with mutated *N-*linked glycosylation sites. In this work we did not seek to systematically study the factors that aid *Plasmodium* expression, seeking instead a “one size fits all” approach to maximize the number of proteins expressed, but minimize the time and resources invested in optimizing expression. However, prior work and our experience suggest that codon optimization is necessary for successful expression of some but not all *Plasmodium* proteins in mammalian expression systems. Previous studies have often used native sequences (reviewed in [102]), even though other studies have noted that optimization of codon usage can aid expression [103,104]. As another example, we have previously found that codon optimization alone had little effect on the expression of PfRh5, while the inclusion of an exogenous signal peptide and the mutation of *N*-linked glycosylation sites significantly increased its expression [50]. In addition, the AT content of the *P*. *vivax* genome (50–60%) is much closer to that of humans (59%) than *P*. *falciparum* (80%). This significantly impacts codon usage between the two *Plasmodium* species (as noted in [105]), and potentially means that *P*. *vivax* proteins would be more easily expressed without codon optimization than *P*. *falciparum* proteins. If expression constructs are to be generated synthetically, as they were in this study, then codon optimization appears to be a useful step, but not a panacea, in the expression of *P*. *vivax* proteins.

To explore whether our library proteins are properly folded, contain conformational epitopes, and are targets for naturally-acquired humoral immunity, we screened 34 *P*. *vivax* recombinant proteins against pooled plasma from 14 Cambodian patients with acute vivax malaria. Of the 34 proteins screened, 27 showed at least a two-fold change in IgG reactivity between naïve sera and the *P*. *vivax*-exposed plasma. Further testing of individual samples may clarify whether some patients show higher IgG reactivity to the proteins with lower reactivity, as IgG responses to *P*. *falciparum* merozoite antigens can vary substantially between individuals [63]. Our results align well with a *P*. *vivax* seroreactivity screen in Korean patients [23], which detected IgG reactivity to 18 full-length proteins and protein fragments, all produced in the wheat germ cell-free system. Of these 18 proteins, seven (MSP1, MSP3.3, MSP10, P12, P41, ARP, and PVX_081550) were represented in our library and all seven showed at least a three-fold change in IgG reactivity between naïve sera and the *P*. *vivax*-exposed plasma in our Cambodian screen. We conclude that these antigens are common targets of immunoreactivity across different transmission regions.

Conformational epitopes were present in 18 of 34 antigens, as indicated by > 20% reduction in IgG reactivity after heat treatment. IgG reactivity to 12 of these 18 antigens was predominantly conformation-specific, as indicated by > 50% reduction in IgG reactivity after heat treatment. These findings suggest that our library proteins are properly folded. The tertiary structure of MSP3 family proteins is known to be recalcitrant to heat denaturation, so the limited change in immunoreactivity following heat treatment of these antigens does not necessarily indicate a lack of tertiary conformation. In addition to MSP3 proteins, several other proteins also show an absent or weak heat-sensitive response, including those with higher overall responses, such as MSP4 and MSP10, and several with weak overall responses, such as P92 and PVX_110965. The lack of heat-sensitive responses may be due to one of three factors, each of which might be applicable for a given protein. First, the protein may not denature or remain denatured under the heat treatment we used (80°C for 10 min), which is likely the case for MSP3. Follow-up experiments testing other techniques, such as chemical denaturation or more extreme heating conditions, could clarify this point. Second, most of the primary antibody response may target linear epitopes within the protein that are not affected by denaturation. At least two of the proteins where responses were not affected by heat treatment, MSP4 and ARP, contain regions of low complexity, which could fit with such a model. Third, the lack of a change in response could indicate an issue with protein quality. Although all of these proteins are visible by western blot, P92 and PVX_001015 are very faint. Additional experiments are needed to fully explore these possibilities, though the fact that the majority of antigens displayed a heat-sensitive response suggests that most library proteins are properly folded.

As well as indicating that the full-length ectodomains contain folded epitopes, these data also indicate that humans naturally acquire IgG responses to multiple *P*. *vivax* proteins, thus supporting their further exploration as candidate vaccine antigens. While our study was not designed to compare the seroreactivity of *P*. *vivax* antigens, five proteins showed high seroreactivity and required a more dilute sera for screening (MSP5, P12, GAMA, CyRPA, and PVX_081550). Future seroreactivity studies using immune sera from *P*. *falciparum*-only endemic areas (e.g., West Africa) are needed to assess the immune cross-reactivity of our *P*. *vivax* recombinant protein library, as well as more in-depth immunoepidemiological studies in various *P*. *vivax*-endemic areas. P12 is a protein of particular interest, as it has shown seroreactivity in 49% of 96 Korean patients with acute vivax malaria [57], and *P*. *falciparum* P12 has shown seroreactivity in 96% of 286 Kenyan individuals [63]. IgG responses to P12 may be a useful marker of infection with *P*. *vivax*, *P*. *falciparum*, or both in broader epidemiological investigations. Cross-sectional studies are needed to determine whether antigen-specific IgG responses correlate with *P*. *vivax* exposure, and to assess their duration following acute malaria episodes. Further prospective studies will be needed to define the magnitude and breadth of IgG responses that confer clinical protection.

We used the protein library to confirm for the first time that the known P12-P41 interaction in *P*. *falciparum* [64] is conserved in the evolutionarily distant but related parasite, *P*. *vivax*. Biophysical measurements using SPR showed that the P12-P41 interaction in *P*. *vivax* appears to be at least three times stronger than in *P*. *falciparum*. Gene knockout experiments have shown that *P*. *falciparum* P12 is not essential for parasite invasion or growth *in vitro*, and antibodies against P12 do not significantly block erythrocyte invasion [64]. The difference in protein-protein interaction affinity between the species indicates that there could be functional differences between them; therefore, we plan to investigate the potential function of the *P*. *vivax* P12-P41 interaction in merozoite invasion of reticulocytes and the possible invasion-blocking effects of immune IgG specific for P12 or P41 in Cambodian *P*. *vivax* isolates *ex vivo*. Interestingly, an interaction between *P*. *vivax* P12 and *P*. *falciparum* P41 was also detected, suggesting the existence of a conserved binding site, which may be an attractive target for vaccines against both *P*. *falciparum* and *P*. *vivax*. The reverse interaction between *P*. *falciparum* P12 and *P*. *vivax* P41 (in both bait-prey and prey-bait orientations) was not detected, which may assist in mapping the P12-P41 binding site within *Plasmodium* species.

Two novel interactions were also detected with AVEXIS and investigated by SPR; both putative interactions were validated by SPR and/or reciprocation of prey-bait and bait-prey interactions in AVEXIS. The failure to detect the *P*. *vivax* MSP3.10-MSP7.1 interaction in the prey-bait orientation by AVEXIS may indicate that artificially pentamerized MSP3.10 prey interferes with the formation of an oligomeric structure necessary for binding. While *P*. *falciparum* MSP3.1 contains a conserved C-terminus important for oligomerization [106], *P*. *vivax* MSP3s are not clear homologs and lack this feature [69,82]. Their potential for forming higher order structures is unknown. SPR showed that the *P*. *vivax* MSP3.10-MSP7.1 interaction has a relatively high avidity, potentially increased due to oligomerization of MSP7.1. Members of the *P*. *vivax* MSP3 family are known to be peripherally associated with the merozoite surface [69] and *P*. *vivax* MSP7s are predicted to have a similar location, based on their *P*. *falciparum* orthologs [70,71]. MSP3 has no known binding partners, and *P*. *falciparum* MSP7 is known to form a complex with MSP1 [70]. The MSP7-MSP1 interaction has not yet been established for *P*. *vivax*; our AVEXIS screen did not detect such an interaction, although only three of the 11 *P*. *vivax* MSP7 family members were included in our library, meaning that one of the members not expressed to date could interact with *P*. *vivax* MSP1. SPR data suggest that the *P*. *vivax* P12-PVX_110945 interaction is extremely weak. PVX_110945 is a hypothetical protein with no known function, but its transcription and genomic location give it a possible function in merozoite development, invasion of erythrocytes, or both. Additional experiments to co-localize these two interacting pairs in parasite isolates may help validate and shed light on the possible biological relevance of these interactions.

We believe that this *P*. *vivax* recombinant library and expression approach will significantly improve our ability to study the biology of *P*. *vivax* erythrocyte invasion and the natural development of *P*. *vivax* immunity. This approach has already been successfully applied to both fronts in *P*. *falciparum* research [50,63,87,88]. Not only will this *P*. *vivax* library enable new screens for parasite-host interactions, it can also contribute to protein structure and immunoepidemiological studies. Structural studies can greatly increase our understanding of the function of these proteins. Such studies can also enhance the possibility of defining protective versus decoy conformational epitopes which may have a tremendous impact on their ultimate potential as vaccine candidates [107]. Future immunoepidemiological studies that use a panel of proteins should enable more systematic comparisons between proteins, in contrast to previous studies that have used one or several proteins. Deposition of our *P*. *vivax* library plasmids in the open plasmid resource, Addgene, should greatly facilitate community efforts to identify, validate, and develop promising vaccines for *P*. *vivax* malaria.

## Supporting Information

S1 Table Plasma samples from Cambodian patients with acute vivax malaria. (XLSX)

S1 Fig P12, P41, and MSP7.1 may exist as homodimers or oligomers. Recombinant his-tagged *P*. *vivax* P12, P41, and MSP7.1 were fractionated by SDS-PAGE under reducing conditions [NuPAGE Sample Reducing Agent (Invitrogen), heated at 70°C for 10 min] and non-reducing conditions, and stained [SimplyBlue SafeStain (Invitrogen)] with an increase in molecular mass upon reduction suggesting the proteins contain disulphide bonds, as expected. No homodimer or oligomers are observed by SDS-PAGE. Samples run under native conditions [NativePAGE Bis-Tris Gel System (Thermo Fisher Scientific), stained with Coomassie Brilliant Blue R-250 (ThermoScientific)], support the presence of homodimers for P12 and P41 with bands near 146 kDa. Monomers for these correspond to 60 and 66 kDa respectively. MSP7.1 shows a faint band near the expected monomer size of 69 kDa and a dominant band near 242 kDa, suggesting that this protein forms an oligomer of potentially three or four monomers.
(TIF)

S2 Fig *P*. *vivax* P12 and *P*. *vivax* P41 show no self-binding by surface plasmon resonance. Increasing concentrations of *P*. *vivax* P12 (A) or *P*. *vivax* P41 (B) were injected over immobilized biotinylated *P*. *vivax* P12 (A) or *P*. *vivax* P41 (B) with no interactions observed.
(TIF)

S3 Fig Surface plasmon resonance supports a weak interaction between *P*. *vivax* P12 and *P*. *vivax* PVX_110945. Increasing concentrations of ***P*. *vivax*** P12 were injected over immobilized biotinylated ***P*. *vivax*** PVX_110945 with weak binding observed. None of the concentrations used reached equilibrium, which prevented the calculation of an equilibrium dissociation constant (***K***_***D***_).
(TIF)
