# Supplementary figures and images for: A Library of Plasmodium vivax Recombinant Merozoite Proteins Reveals New Vaccine Candidates and Protein-Protein Interactions
Source: PLoS Negl Trop Dis. 2015 Dec 23;9(12):e0004264. doi: 10.1371/journal.pntd.0004264 (PMC4689532; doi:10.1371/journal.pntd.0004264)

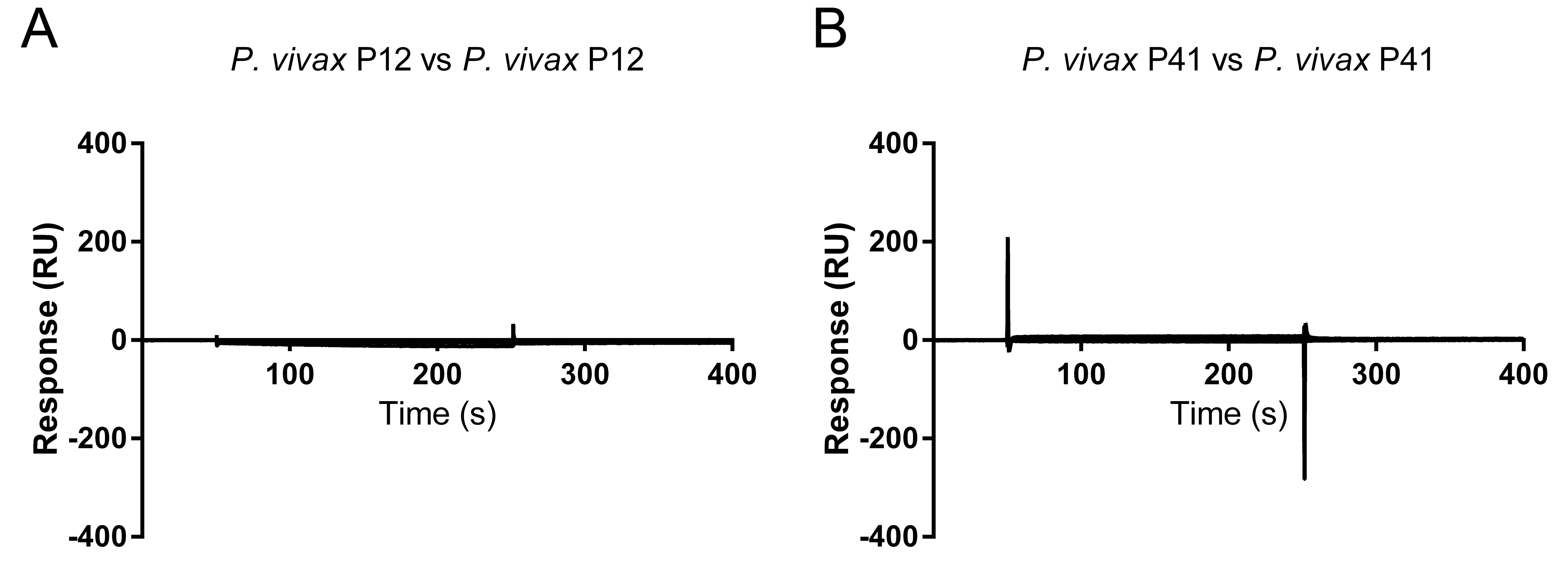

Supplement: S2 Fig — Increasing concentrations of P. vivax P12 (A) or P. vivax P41 (B) were injected over immobilized biotinylated P. vivax P12 (A) or P. vivax P41 (B) with no interactions observed. (TIF) [file pntd.0004264.s003.tif]

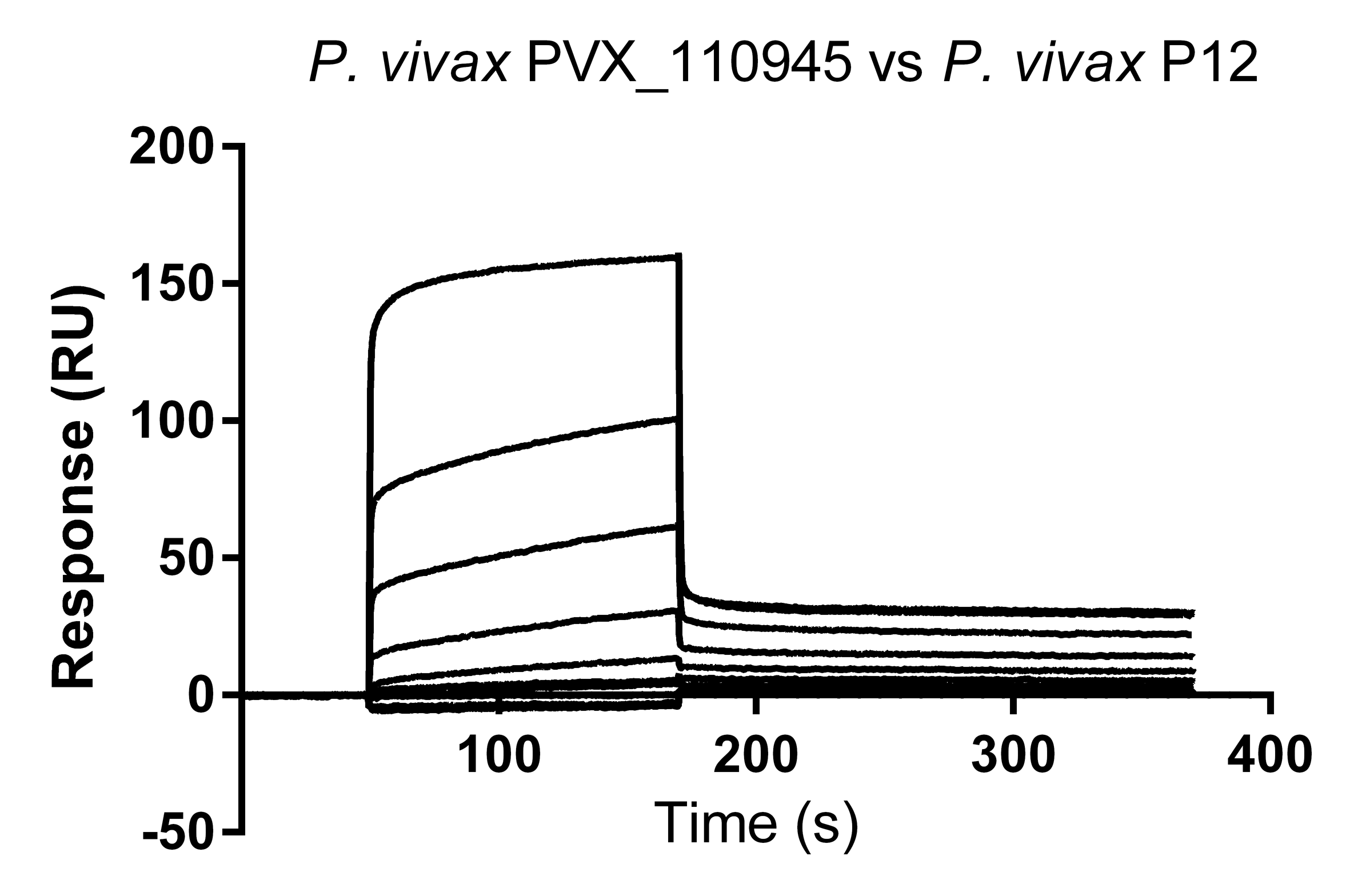

Supplement: S3 Fig — Increasing concentrations of P. vivax P12 were injected over immobilized biotinylated P. vivax PVX_110945 with weak binding observed. None of the concentrations used reached equilibrium, which prevented the calculation of an equilibrium dissociation constant (K D). (TIF) [file pntd.0004264.s004.tif]
